# The Medical Surveillance Monthly Report: The First 30 Years

**Published:** 2025-04-20

**Authors:** Leslie L. Clark, Mark V. Rubertone

In April 1995 the inaugural issue of the *Medical Surveillance Monthly Report* declared, “If the *MSMR* is not useful to its readers, it will have no value.” ^1^ Throughout its 30-year history, *MSMR* has continuously sought to improve its content with the ultimate goal of providing its readers with unbiased, scientifically rigorous, evidence-based information on the current status, trends, and determinants of the physical and mental health of U.S. military service members. Empowering military public health leaders with timely access to militarily relevant routine and specialized reports positions them to identify and contain outbreaks, understand disease burden, guide policy changes, and evaluate and improve prevention and control strategies. *MSMR* 's utmost priority is publishing articles and summary data directly relevant to the health, safety, well-being, and military operational fitness of the members of the U.S. military.

On the first page of the first issue of *MSMR* , executive editor John Brundage, MD, MPH, articulated the new journal's objectives as “medical surveillance information of broad interest…The ultimate goal…is to provide…information necessary to inform, motivate, and empower commanders, their surgeons, and medical staffs to design, implement, and resource programs that enhance health, fitness, and readiness.” ^1^

The need for a publication like *MSMR* was evident in the early 1990s due to the lack of dissemination of routine periodic medical surveillance in the U.S. military, exacerbated by the cessation of publication of service-specific surveillance reports including *Health of the Army* and *Statistics of Navy Medicine* in the late 1980s. In addition, at the time there were no ready nor centrally available sources of timely and reliable information on extant medical threats, and published insights on medical situational awareness were generally out of date, incomplete, and largely uninformative. In its formative years, one of *MSMR* 's core functions was to report routine monthly surveillance statistics not otherwise readily available to intended readership.

*MSMR* was also intended to emulate, for the U.S. military, the *Morbidity and Mortality Weekly Report (MMWR)* published by the U.S. Centers for Disease Control and Prevention (CDC). Like *MMWR, MSMR* is a mechanism to disseminate public health data and reports targeted principally to military public health professionals, in addition to military commanders, leaders and policy-makers, as well as the scientific and lay press. Dissemination is a core function of public health surveillance, defined by the CDC as “the ongoing, systematic collection, analysis, and interpretation of health data, essential to the planning, implementation and evaluation of public health practice, closely integrated to the dissemination of these data to those who need to know and linked to prevention and control.” ^2^

A key difference between civilian and military public health surveillance is the military's focus on force health protection and medical readiness, along with communication of health threats to military commanders. ^3^ This focus has driven *MSMR* 's desire to provide unbiased, scientifically rigorous, and evidence-based estimates of the incidence, distribution, impact and trends of illness, injury, and other health threats to the physical and mental health of U.S. military members, as well as drawing attention to conditions that are “high burden” for the military and have an associated effect on the health of the force.

*MSMR* represented one of the first and most widely visible products of the U.S. Army Center for Health Promotion and Preventive Medicine (USACHPPM). Initially, *MSMR* primarily reported Army-specific surveillance summaries of hospitalizations; notifiable diseases (i.e., reportable medical events); counts, rates, and trends of illnesses and injuries of surveillance interest (e.g., acute respiratory illness, sexually transmitted infections, heat and cold related injuries); and field reports of outbreaks and medical events of interest to military medical staffs and commanders. *MSMR* , however, rapidly grew and evolved prompted both by increases in the type and sources of data available for analyses, as well as a desire to provide more complex analyses to help interrogate threats to the health of the force.

During *MSMR* 's relatively short lifespan, the military health data infrastructure has grown in extraordinary ways. These three decades have produced remarkable advancements in comprehensive notifiable disease reporting, expansion of deployment-related health care, and more complete capture of health care provided to military service members and beneficiaries, including incorporation of prescription drug data, laboratory tests and results, immunizations, mortality data, and an extensive array of periodic and time-sensitive health assessments.

When *MSMR* began publishing in 1995, comprehensive and reliable health surveillance data for all services were not routinely transmitted nor stored in a centralized repository, as they were for the U.S. Army in USACHPPM's newly established Army Medical Surveillance Activity (AMSA). By the following year, however, AMSA had begun receiving monthly personnel rosters of all members of all services, retroactively to 1990. In 1997, this comprehensive database transitioned into the Defense Medical Surveillance System (DMSS) and began routine receipt of health surveillance data from sources throughout the Department of Defense (DOD). These data were critical for ascertaining and calculating timely and accurate counts, rates and trends in illness and injury for all members of all services.

**Figure:**
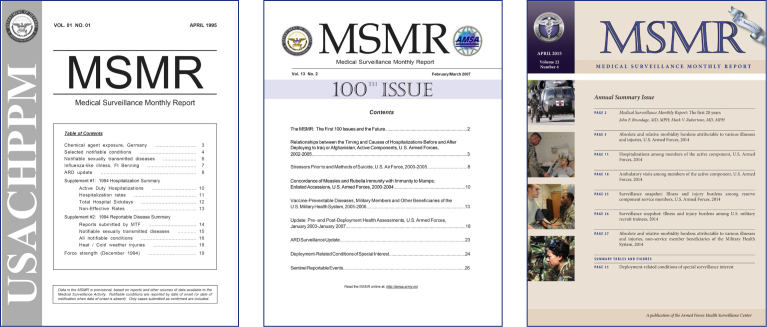


This evolution in health surveillance reporting led to the creation, in 2002, of the first *MSMR* report evaluating the morbidity burden of illnesses and injuries to the U.S. Armed Forces: “Relative Burdens of Selected Illnesses and Injuries, U.S. Armed Forces, 2001.” Using a modification of the classification system developed by the Global Burden of Disease Study, ^4^ the report and its accompanying data tables provided a means of summarizing the annual numbers of medical encounters, hospital bed days, and unique individuals affected using the inpatient, outpatient, and personnel records available in the DMSS. This report evolved into *MSMR* 's annual issue that provides updated summaries of all hospitalizations, outpatient visits, medical evacuations, deployed medical care, and morbidity burdens of illnesses and injuries among members of the U.S. Armed Forces, as well as non-service member beneficiaries of the Military Health System (MHS).

The annual burden of health care issue highlights an example of *MSMR* analyses that presage issues of military medical importance. Well before post-traumatic stress disorder (PTSD) and traumatic brain injury (TBI) were recognized as “signature wounds” of the wars in Afghanistan and Iraq, *MSMR* was highlighting the importance of mental disorders, including mood disorders and adjustment reactions, and musculoskeletal injuries, including injuries of the head and neck, as major sources of morbidity, lost duty time, and health care use among military members. ^5^

Like the rest of the U.S. military, *MSMR* was challenged to respond to the events of September 11, 2001, and the war that ensured over the following decade. In response, *MSMR* initiated reports documenting illnesses including heat and cold injuries, PTSD, malaria, and leishmaniasis, as well as injuries such as traumatic amputations and traumatic brain injuries associated with service in combat zones.

The February / March 2007 edition of *MSMR* marked its 100th issue, and through its initial 12 years of publication, *MSMR* had disseminated approximately 240 reports of surveillance findings and results of preventive interventions; 50 reports of outbreaks, of which approximately 80% were on infectious diseases; and 40 case and case series reports, of which approximately 85% were on infectious diseases. ^6^ The editorial leading the 100th issue milestone highlighted the “…steady stream of unimaginable events with profound military medical significance” since the publication of the first issue:

…including the initiation and conduct of U.S. military operations in the Balkans; terrorist attacks on the United States (including the Pentagon) on 11 September 2001; the initiation and conduct of the global war on terrorism; widespread uses of vaccines for military-specific indications, including smallpox, anthrax, and tick-borne encephalitis; outbreaks of ‘mysterious’ illnesses with unknown causes among deploying / deployed U.S. troops; life-threatening hyponatremia from excessive water consumption in heat stressful conditions; the reemergence of vivax malaria along the demilitarized zone in Korea; the loss of vaccines against adenovirus types 4 and 7—and the reemergence of adenoviruses as significant causes of acute respiratory disease among military recruits; interrupted supplies of benzathine penicillin for preventing severe group A beta hemolytic streptococcal diseases among recruits; uses of the DOD Serum Repository for health surveillance, policymaking, and medical research purposes; outbreaks of community-acquired methicillin-resistant *S. aureus* (MRSA), particularly among recruits; routine health assessments before and after overseas deployments; numerous combat casualties, illnesses, and non-battle injuries during service in Afghanistan and Iraq, including wounds from conventional and improvised munitions, accidents, and endemic and nosocomial infections (e.g., leishmaniasis, malaria, multiple drug resistant *Acinetobacter baumanii* ); greater appreciation of the scopes and consequences of post-traumatic stress reactions and emerging infections; and many others. ^6^

The 100th issue of *MSMR* also fore-shadowed its coming evolution as the publication of record for the Armed Forces Health Surveillance Center (AFHSC), the precursor of today's Armed Forces Health Surveillance Division (AFHSD). The AFHSC was established by the Deputy Secretary of Defense in 2008 ^7^ through the combination of the resources of legacy organizations AMSA, the DOD Global Emerging Infectious Disease Surveillance and Response System (DoD-GEIS), and the Global Health Surveillance Activity supporting the Force Health Protection Directorate in the Office of the Assistant Secretary of Defense for Health Affairs. AFHSC was charged with promoting, maintaining, and enhancing the health of U.S. military and military-associated populations, through relevant, timely, actionable, and comprehensive health surveillance information. ^7^ The establishment of the AFHSC represented a consolidation of DOD efforts to improve health surveillance capabilities throughout all services.

*MSMR* 's rapid evolution during this time included a broader scope concurrent with its new emphasis on all services, while continuing to publish surveillance analyses on topics that were militarily important, timely, and relevant. Subject areas with high priority for *MSMR* attention included health threats associated with military training and operations; effects of force health protection measures; and other specific concerns of military members and their families, advocacy groups, politicians, the popular press, and others. *MSMR* 's focus on deployment health issues sharpened during periods of high operational tempo.

To emphasize the potential impacts of MSMR's published surveillance data and new findings on force health protection and readiness, MSMR reformatted its layout in November 2018, introducing new text boxes for full reports that briefly summarize their new findings—“What are the new findings?”—in addition to placing those findings in context—“What are the implications for force health protection?”—following the general abstract.

The creation of the AFHSC and the continued development of its extensive data warehouse, DMSS, with its broad analytic capabilities, facilitated *MSMR* 's ability to provide routine surveillance statistics regularly for a wide variety of health leaders and epidemiologists. *MSMR* content continued its expansion to include in-depth surveillance analyses pertaining to diverse populations, trends over multi-year periods, and risk factors for diseases and injuries of particular interest. Because readers and the combatant commands expressed interested in topics such as hospital-acquired infections, dental readiness, physical fitness data, for example, not able to be addressed using DMSS databases alone, *MSMR* encouraged more submissions from outside sources with access to other data sets or responsibility for disease and injury prevention research or epidemiological investigations. Additional changes included a new appearance, more widespread distribution, and improved accessibility via a new website. ^6^

In 2011, *MSMR* applied and was accepted for indexing in MEDLINE, the principal online bibliographic citation database of the National Library of Medicine's MEDLARS _®_ system. The acceptance of *MSMR* for indexing in MEDLINE validated its evolution and development as an evidence-based peer-reviewed journal. To be accepted to MEDLINE, *MSMR* was evaluated on its scientific policy and quality, and found to have sufficient merit for inclusion in the database. This independent designation formally distinguished *MSMR* content as fundamentally different from routine reports or *ad hoc* requests produced by AFHSD. It also further expanded the scope and reach of its content and increased the number and quality of external submissions to *MSMR* .

The establishment of the Defense Health Agency (DHA) in 2013 formally consolidated the medical services of all branches of the U.S. military, which included integration of all U.S. military public health surveillance activities. These integration efforts reinforced *MSMR* 's focus on reporting results for all service branches. As a result, *MSMR* established an editorial advisory board of leaders from all military services. The advisory board continues to be a key part of *MSMR* 's continuous quality improvement efforts and an important element of ensuring key stake-holder involvement and input.

Two years later, *MSMR* 's April 2015 issue marked its 20th anniversary. The editorial leading that issue highlighted several elements that were instrumental in its progress to that point, including “unprecedented support of military force health protection and health surveillance initiatives and unimaginable advances in telecommunications and information management/data warehousing technologies.” ^8^

Over the past decade, *MSMR* has continued to explore ways to expand and improve its content and make it more readily usable to readers. *MSMR* increased its production of thematic issues and made significant efforts to engage subject matter experts throughout the MHS, for submissions of reports on thematic issues in addition to invited editorials that contextualized surveillance findings. These thematic issues have focused on a wide range of subjects including women's health, mental and behavioral health, heat- and cold-related injuries and illnesses, sexually transmitted infections, gastrointestinal infections, vision-related conditions, and a Global Emerging Infections Surveillance (GEIS)-themed issue with surveillance reports from GEIS partners.

A review published in the January 2024 issue of *MSMR* ^9^ summarized the journal's content over the preceding 5 years and presented areas of interest for future *MSMR* submissions including, but not limited to, topics related to improving biodefense posture consistent with the 2023 DOD Biodefense Posture Review; submissions in the area of pharmacoepidemiology, utilizing the data from the Pharmacy Data Transaction Services (PDTS); and outbreak and field reports, primarily with significance beyond the setting in which they occurred. This review also lists the 10 most-read articles on the *MSMR* website during the 5-year period. Notably, 2 of the most-read articles—on heat injury and routine screening for antibodies to HIV—represent reports that were some of the earliest developed by *MSMR* and published annually. Also significant is that 3 of the articles were co-authored by military preventive medicine residents during their rotations at the AFHSD, highlighting a little known but valuable synergy with preventive medicine residency training at the Uniformed Services University for Health Sciences.

*MSMR* staff has contributed further significant value to the MHS in the development and dissemination of standardized case definitions for health surveillance. *MSMR* editorial staff, in consultation with other AFHSD epidemiologists and other MHS subject matter experts, has helped develop over 100 standardized case definitions designed for use with administrative health care data derived from the U.S. military electronic health record and contained in the DMSS and other available datasets. Many (although not all) of these case definitions are readily accessible to other public health and epidemiological researchers via the surveillance case definition website on the AFHSD Epidemiology and Analysis website. ^10^ Case definitions are regularly reviewed and updated by the Surveillance Methods and Standards (SMS) Working Group of the AFHSD. This provides a valuable resource that furthers the goal of increasing standardization in surveillance methods and practices throughout the DHA.

The future of *MSMR* will undoubtedly benefit from increasingly modernized public health data infrastructure and data analysis and integration capabilities. Unprecedented access to this extensive and expanding network of data, along with advanced forecasting and data analytics, will allow *MSMR* to continue its longstanding role in providing timely access to reports on population-based morbidity, risk assessments, vaccine adverse effects, emerging threats, deployment surveillance, policy effects, serological surveys, and sero-epidemiological research. ^11^ *MSMR* analyses are regularly referenced in reports developed for and used by governmental agencies to inform policy-makers throughout the U.S. Government, including the Congressional Research Service ^12 - 14^ and the United State Government Accountability Office, ^15^ demonstrating *MSMR* 's utility and reach as a readily accessible, accurate, and useful source of health surveillance information.

**Figure:**
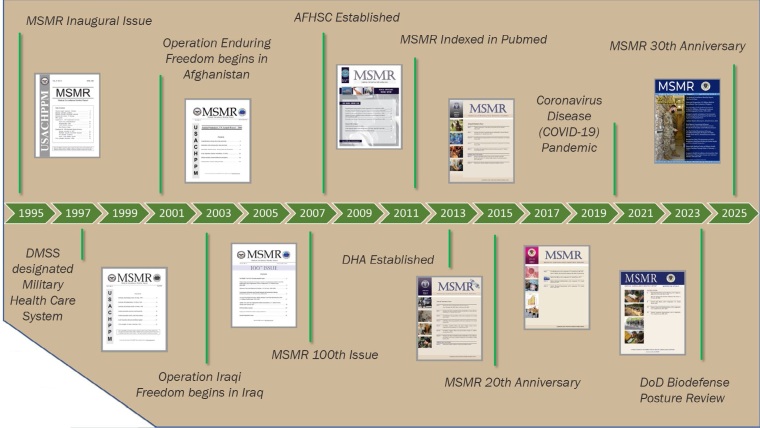
The First 30 Years of MSMR

As *MSMR* enters its 31st year, its editorial staff aims to continue its tradition of excellence while making its content more clinically relevant, continuing to increase collaboration with external agencies and individuals, publishing topics of military relevance, and making practical military-specific recommendations based on sound scientific evidence.
